# Restrained Eating Features and Brain Morphology: A Pediatric Population‐Based Study

**DOI:** 10.1002/eat.24445

**Published:** 2025-04-26

**Authors:** C. P. M. Steegers, M. E. J. Deen, P. W. Jansen, T. White, K. F. M. Bracké, M. H. J. Hillegers, G. C. Dieleman

**Affiliations:** ^1^ Department of Child and Adolescent Psychiatry/Psychology Erasmus MC‐Sophia Children's Hospital Rotterdam the Netherlands; ^2^ The Generation R Study Group Erasmus University Medical Center Rotterdam the Netherlands; ^3^ Department of Psychology, Education, and Child Studies Erasmus University Rotterdam Rotterdam the Netherlands; ^4^ Department of Radiology and Nuclear Medicine Erasmus MC Rotterdam the Netherlands; ^5^ Section on Social and Developmental Cognitive Neuroscience National Institute of Mental Health Bethesda Maryland USA

**Keywords:** anorexia nervosa, body‐mass index, brain volume, children, eating disorders, general population, neuro‐imaging, restrained eating, structural MRI

## Abstract

**Objective:**

Anorexia nervosa, a restrictive eating disorder that is most commonly seen in females, is associated with alterations in gray matter (GM) and white matter (WM) structures. However, little is known about how restrained eating (RE) and the body mass index‐standard deviation score (BMI‐SDS) are related to brain morphology and whether sex differences exist in the general pediatric population.

**Method:**

Participants were 9‐year‐old girls and boys (*n* = 2729) from the population‐based Generation R Study. BMI‐SDS was calculated by adjusting BMI for sex and age, using Dutch growth curves. RE is measured with the Dutch Eating Behavior Questionnaire. All children underwent structural magnetic resonance imaging, and brain volumes were calculated using FreeSurfer.

**Results:**

BMI‐SDS in girls was positively associated with total intracranial volume and several regional brain volumes. In addition, BMI‐SDS showed an inverted U‐shaped association with total GM and WM. In girls, RE had a positive linear association with total intracranial, WM, and several regional brain volumes, corrected for BMI‐SDS. Additionally, there was an inverted U‐shaped association with the amygdala and insula volume. In boys, we found merely positive linear associations between BMI‐SDS and brain volumes, and no associations between RE and brain volumes.

**Discussion:**

Associations between BMI and brain volumes exist in typically developing children, but there are significant sex differences in the magnitude and shape of the associations. RE is associated with some differences in brain volumes in girls only. Longitudinal studies are needed to assess these associations over a longer period of time.

**Summary**

- This research is about a large group of healthy girls and boys and whether there is a difference in certain brain structures in children with lower or higher body weight or in children who eat less than desired (restrained eating).
- We found that, in girls, both body weight and restrained eating are associated with specific larger and/or smaller brain structures, while in boys, only body weight is linked to the size of specific brain structures.

## 1 Introduction

Anorexia nervosa (AN) is a severe restrictive eating disorder that often begins in adolescence or young adulthood, with a predominant female prevalence. AN is characterized by a severe distortion in body image coupled with an intense fear of gaining weight, or persistent behavior that interferes with weight gain, even at a significant low weight, even though this fear is no longer required by the DSM‐5 (American Psychiatric Association 2013). These distortions can drive life‐threatening weight loss, resulting in high morbidity and mortality rates (Papadopoulos et al. 2009). The etiology is multifactorial, including psychological, social, and neurobiological factors (Zipfel et al. 2015). Structural magnetic resonance imaging (MRI) techniques are used to unravel the biological underpinnings of AN, which are not yet fully elucidated. A better understanding of the underlying disease mechanisms may help to predict the course of AN (Bracké et al. 2023) and develop more effective treatments. The first step in studying neural underpinnings through neuroimaging is examining global brain metrics, such as global gray matter (GM) and white matter (WM) volume, to screen for large alterations and to provide direction on which brain regions may be relevant to investigate further. The GM regions of the brain contain a diverse array of neuronal cells and abundant connections between these cells. GM is traditionally considered to be involved in the computation and processing of information within the brain. Brain WM represents myelinated nerve fibers and provides rapid, long‐distance communication between brain regions. The outer layer of the cerebral cortex involves a layer of GM that is approximately 2‐ to‐ 5 mm thick. The thickness of this outer layer can be measured across the brain and reflects the cortical thickness at any cortical region of the brain. Finally, the human brain is not completely smooth but contains a pattern of fissures and folds. This folding pattern emerges during prenatal life in a process called gyrification of the brain. The degree of folding patterns in the brain can be quantified and is described as brain gyrification.

A review of structural MRI studies in individuals with AN shows striking reductions in global WM and GM volumes, including cortical thickness and gyrification, and reduced local GM volumes during the acute phase of the disorder. The GM loss in these local brain areas is most pronounced in the hippocampus, prefrontal, parietal, and temporal cortex, amygdala, insula, thalamus, and cerebellum (Kappou et al. 2021). Compared to adults with AN, GM volumes appear to be more reduced in adolescents with AN (Seitz et al. 2014, 2016). Conversely, some studies report increased rather than reduced GM volumes in certain brain areas in females with AN, including the medial orbitofrontal (mOFC) and dorsolateral orbitofrontal cortex (lOFC) and the right insula (Seitz et al. 2016; Frank 2015). As these dramatic brain changes in the acute phase of the disorder are at least partially, and often completely, reversible with refeeding (King et al. 2015; Lázaro et al. 2013; Bernardoni et al. 2016; Walton et al. 2022), they likely reflect consequences of malnutrition rather than providing clues on the underlying etiology (Frank 2015). The cerebral structures affected in individuals with AN are components of the limbic system and the reward network. Distinct emotional and perceptual neural circuits have been hypothesized to underpin symptoms of AN, including how individuals perceive and respond to food and feelings of hunger, experience body image, and emotional cues (Lipsman et al. 2015; O'Hara et al. 2015). Furthermore, preliminary evidence suggests that the cerebral structures implicated in AN are also involved in regulating eating behavior in healthy individuals (Smeets et al. 2012).

There exists a paucity of research on restrained eating (RE), low body‐mass index standard deviation score (BMI‐SDS) and brain morphology in the general pediatric population. Nonetheless, further research might assist in a better understanding of associations and the possible etiology of eating disorders. In a previous study within the same population as the current study, we found an inverted U‐shaped association between BMI‐SDS and global gyrification in both boys and girls (Steegers et al. 2021). This finding is partly corroborated by the results of Alosco et al., who reported a negative association between BMI‐SDS and total GM volume in 120 typically developing boys and girls between the ages of 6 and 18 years. No association was found between BMI‐SDS and WM volume (Alosco et al. 2014). Kennedy et al. documented a negative association of BMI‐SDS with GM and WM volumes in several brain regions associated with reward and control processing and sensory integration in 137 adolescents with a moderate to high BMI (Kennedy et al. 2016). And finally, in the large ABCD study (*N* = 11,875), an inverted U‐shaped association of BMI‐SDS with total brain volume (TBV) and volumes of the hippocampus, anterior cingulate cortex, and lOFC is found (Bohon and Welch 2021). However, none of these studies included the relationship between RE and brain morphology.

In view of the fact that cases of AN are predominantly diagnosed in females, the majority of neuroimaging studies have been conducted in female patients with AN. The present study, therefore, has the objective of investigating associations between RE, BMI‐SDS, and brain morphology in both girls and boys from the general population. Based on findings from previous AN research, we hypothesize that BMI‐SDS and RE in middle childhood are negatively associated with cerebral GM volumes in girls. In light of the non‐linear associations between BMI‐SDS and gyrification that are previously identified in our research (Steegers et al. 2021), yet the absence of exploration into the relationship with eating behaviors, the present study is undertaken to replicate and extend the aforementioned association between BMI‐SDS and RE, and to elucidate its correlation with GM volumes. In our exploratory analyses, we, therefore, examine regions commonly affected in AN, including the amygdala, cingulate cortex, hippocampus, lOFC, mOFC, and insula, which play a role in emotion regulation, learning, decision‐making, and cognitive control.

## 2 Method

### 2.1 Design

This study was embedded in the Generation R Study, which is a population‐based prospective cohort from fetal life onward (Kooijman et al. 2016). Pregnant women who were living in Rotterdam, the Netherlands, with an expected delivery date between April 2002 and January 2006 were invited to participate (participation rate: 61%). After birth, children completed multiple assessment waves (Jaddoe et al. 2012). The current study used data collected at a mean age of 9.7 years for the children. Written informed consent was obtained from all participating children and their legal representatives. The study was approved by the Medical Ethics Committee of the Erasmus University Medical Center, Rotterdam.

### 2.2 Participants

**FIGURE 1:**
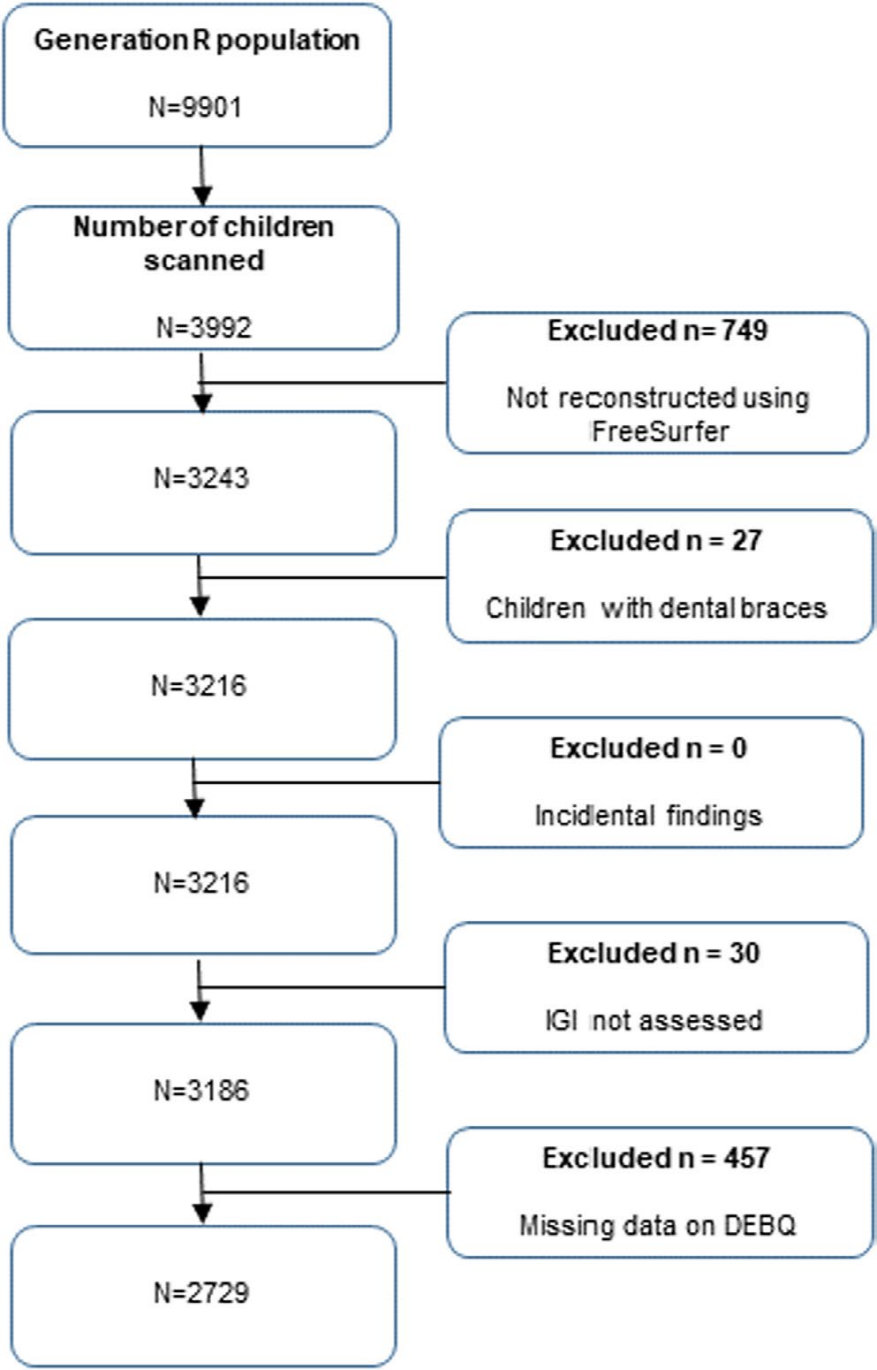
Flowchart inclusion participants. DEBQ = Dutch eating behavior questionnaire; LGI = local gyrification index.

The baseline population consisted of 9901 live‐born children and their parents. Those without MRI or RE data (*n* = 5909 and 457, respectively) and those with poor quality MRI data (*n* = 806) (see Figure 1 for the flow diagram) were excluded, leaving a final sample consisting of 2729 participants.

### 2.3 Measures

#### 2.3.1 BMI‐SDS

Child height and weight were measured in a dedicated research center by trained staff (Jansen et al. 2012). Standing height was measured using a stadiometer (Holtain Limited). Weight was measured in light clothing using an electronic scale (SECA). BMI‐SDS was calculated by adjusting BMI (kg/m^2^) for sex and age, using Dutch growth curves (Schönbeck et al. 2011).

#### 2.3.2 Restrained Eating

At around 9 years of age, the child's RE was measured with the Restrained Eating scale of the validated parent‐report version of the Dutch Eating Behavior Questionnaire (DEBQ) (Braet and Van Strien 1997). The scale consisted of nine items that assess RE and the intention to eat less than desired in order to lose or maintain body weight. Child eating behavior was rated using a five‐point Likert scale from 1 = *never* to 5 = *always*. In the Generation R Study, a slightly adapted version of the DEBQ was used, in which one item (eating in the evening) was omitted, since Dutch 9‐year‐olds generally do not eat much in the evening. The Cronbach alpha was 0.90, indicating a good internal consistency.

#### 2.3.3 MRI Data Acquisition

MRI images were acquired with a 3.0 Tesla GE 750w MR system (General Electric Healthcare, Milwaukee, WI, USA) using an eight‐channel head coil. *T*_1_‐weighted structural images were acquired with an inversion recovery‐prepared fast spoiled gradient recalled sequence (TR = 8.77 ms, TE = 3.4 ms, TI = 600 ms, NEX = 1, flip angle = 10°, field of view = 220 × 220 mm, number of slices = 230, slice thickness = 1.0 mm^3^) (White et al. 2018). To assess scanner stability, we performed a resting state sequence on an agar phantom every morning. In addition, a phantom scan was performed once a week to measure geometric distortion (White et al. 2018). Before the actual MRI, the children underwent a mock scanning session to familiarize them with the procedure (White et al. 2013).

#### 2.3.4 Image Processing and Quality Assessment

*T*_1_‐weighted images were processed using FreeSurfer software, version 6.0. (http://surfer.nmr.mgh.harvard.edu). The technical details of these procedures were described elsewhere (Muetzel et al. 2019). Briefly, these include motion correction and averaging of *T*_1_‐weighted images, removal of the non‐brain tissue, Talairach transformation, segmentation of the WM and GM structures, tessellation of the GM‐WM boundary, topology correction, and surface deformation to identify the GM‐WM boundary and the GM‐cerebrospinal fluid boundary. Before the surface‐based analysis, the image was smoothed using a 10 mm full‐width‐at‐half‐maximum Gaussian kernel. After preprocessing with FreeSurfer, images were visually inspected to assess whether white and pial surfaces followed correct boundaries. Subcortical segmentations were visually inspected for each scan according to the ENIGMA structural imaging quality checking protocol (http://enigma.ini.usc.edu/). Subcortical volumes were calculated using the FreeSurfer automated segmentation procedure.

### 2.4 Covariates

Several covariates were selected, based on the literature showing associations with brain morphology and with BMI‐SDS (Pfefferbaum et al. 2016; Vijayakumar et al. 2016; Ritchie et al. 2018; Ducharme et al. 2016; Brito and Noble 2014; Donald et al. 2015). Covariates included maternal alcohol consumption during pregnancy and maternal education level. Furthermore, as genetic differences between ethnic groups may influence brain development (Pfefferbaum et al. 2015) and ethnicity is also related to obesity risk (Endalifer and Diress 2020), ethnicity was included as a covariate (Dutch, Western and Non‐Western). The analyses including the specific brain regions (amygdala, cingulate cortex, hippocampus, lOFC, mOFC, and insula) were also adjusted for intracranial volume to test whether associations were independent of brain size.

### 2.5 Statistical Analyses

Statistical analyses were performed using SPSS version 25.0.0.1. First, the population characteristics of our sample were described, stratified by sex (Table 1), given the large sex imbalance in AN.

**TABLE 1:** Characteristics of 2729 generation R participants included in this study.

Characteristics	Statistic	Girls		Boys	
		*N*	Percentage, median (IQR) or mean (SD)^a^	*N*	Percentage, median (IQR) or mean (SD)^a^
Age at DEBQ assessment (years)	Mean, SD	1374	9.70 (0.28)	1355	9.71 (0.28)
Age at BMI assessment (years)	Mean, SD	1374	9.76 (0.28)	1354	9.78 (0.30)
Age at MRI assessment (years)	Mean, SD	1374	10.01 (0.55)	1355	10.14 (0.59)
Ethnicity		1356		1348	
Dutch	Percentage	891	64.8	879	64.9
Other Western	Percentage	131	9.5	101	7.5
Non‐Western	Percentage	334	24.3	368	27.2
BMI‐SDS*	Mean, SD	1372	0.18 (1.00)	1355	0.27 (1.00)
Restrained eating score (DEBQ)**	Median, IQR	1374	11.00 (9.00–15.00)	1355	10.00 (9.00–14.00)
Brain volumes					
Total intracranial volume (mm^3^)**	Mean, SD	1374	1.45 × 10^6^ (1.15 × 10^5^)	1355	1.59 × 10^6^ (1.27 × 10^5^)
Total gray matter volume (mm^3^)**	Mean, SD	1374	7.33 × 10^5^ (5.35 × 10^4^)	1355	7.97 × 10^5^ (5.73 × 10^4^)
Total cerebral white matter volume (mm^3^)**	Mean, SD	1374	4.03 × 10^4^ (4.06 × 10^4^)	1355	4.51 × 10^5^ (4.51 × 10^4^)
Amygdala (mm^3^)**	Mean, SD	1374	1704.02 (163.56)	1355	1855.09 (184.05)
Cingulate (mm^3^)**	Mean, SD	1374	12,102.00 (1351.52)	1355	13,204.38 (1474.36)
Hippocampus (mm^3^)**	Mean, SD	1374	3910.48 (328.64)	1355	4169.20 (357.18)
Lateral orbitofrontal (mm^3^)**	Mean, SD	1374	9199.07 (978.60)	1355	9903.47 (1037.80)
Medial orbitofrontal (mm^3^)**	Mean, SD	1374	6219.88 (685.01)	1355	6778.31 (751.26)
Insula (mm^3^)**	Mean, SD	1374	11,778.63 (1175.95)	1355	12,864.98 (1316.82)
Age of the mother	Mean, SD	1374	31.32 (4.69)	1355	31.61 (4.65)
Education level of the mother^b^		1284		1277	
High	Percentage	719	52.3	735	54.2
Medium	Percentage	504	36.7	476	35.1
Low	Percentage	61	4.4	66	4.9
Maternal alcohol consumption during pregnancy		1163		1123	
Never	Percentage	457	33.3	388	28.6
Until pregnancy	Percentage	175	12.7	161	11.9
Continued occasionally	Percentage	411	29.9	465	34.3
Continued frequently	Percentage	120	8.7	109	8.0

Abbreviations: BMI‐SDS, body mass index–standard deviation score; DEBQ, Dutch Eating Behavior Questionnaire for children; IQR, interquartile range; SD, standard deviation.

* Significant difference between boys and girls (*p* < 0.05).

** Significant difference between boys and girls (*p* < 0.01).

^a^ Values are percentages for categorical variables, medians (IQR) for continuous non‐normally distributed variables and means (SD) for continuous normally distributed variables.

^b^ High: higher vocational education and higher academic education; Medium: lower vocational training; Low: ranging from no education to high school level.

Pearson correlations between all variables were calculated and are provided in Tables S1 and S2. The RE scale of the DEBQ was left‐skewed. Log transformation did not change the distribution. Therefore, the RE scale was analyzed as a continuous variable considering the large sample size (Field 2016).

To examine the association of BMI‐SDS with brain morphology, we performed a series of linear regression analyses in girls and boys separately, with BMI‐SDS as the determinant and the following brain volumes as separate outcomes: TBV, total GM volume, total WM volume, amygdala, cingulate cortex, hippocampus, lOFC, mOFC, and insula. Analyses were adjusted for the aforementioned covariates. Regression analyses with BMI‐SDS as the determinant and total GM and total WM volume were repeated to add to the perspective and readability of the analyses (Steegers et al. 2021). Then we repeated the above analyses with RE as the determinant and the same brain volumes as separate outcomes. These analyses were also adjusted for the covariates, and in an additional step for BMI‐SDS, given that RE and BMI‐SDS correlated moderately (girls: *r* = 0.46, *p* < 0.01; boys: *r* = 0.42, *p* < 0.001; see Tables S1 and S2). In each analysis, we investigated whether a linear or quadratic association best fit the data (Table S3).

To test the robustness of the significant results, we performed analyses of covariance (ANCOVAs) by dichotomizing BMI‐SDS and RE in sensitivity analyses. For quadratic models, a median split was performed on both determinants. Linear regression analyses were performed with brain volumes as separate outcomes. For linear models, a split was performed on the 20% lowest BMI‐SDS and 80% highest RE. ANCOVAs were then performed with brain volumes as separate outcomes.

Analyses were adjusted for multiple testing using the Benjamini–Hochberg approach, controlling for the false discovery rate at *q*‐value = 0.05 (Benjamini and Hochberg 1995). The procedure was performed separately for girls and boys, correcting for 18 analyses in each group (2 determinants [RE and BMI‐SDS] times 9 brain outcomes). Missing values on the covariates (0% for sex, child and maternal age, 0.01% for ethnicity, 0.001% for BMI‐SDS, 0.06% for maternal education and 16.3% for maternal alcohol consumption during pregnancy) showed no specific pattern and were therefore assumed to be missing at random. Therefore, multiple imputation was performed using the Markov Chain Monte Carlo method, creating 5 imputed datasets with 10 iterations to estimate missing values in the covariates. Pooled results are presented. Adjusted *R* ^2^ change coefficients based on the original data were calculated to estimate the percentage of variation explained by the independent variables. Effect sizes are reported as *R* ^2^.

## 3 Results

### 3.1 Nonresponse Analysis

Excluded children with missing data on the DEBQ (*N* = 457) were compared with included children (*N* = 2729). Data were more frequently missing in children with a higher BMI‐SDS (*t* = 3.94, df = 571.15, *p* < 0.001) and with smaller brain volumes (TBV: *t* = −5.03, df = 3184, *p* < 0.001; total GM volume: *t* = −6.31, df = 3184, *p* < 0.001; total WM volume: *t* = −4.0, df = 3184, *p* < 0.001; amygdala: *t* = −2.98, df = 3184, *p* < 0.001; cingulate: *t* = −5.91, df = 3184, *p* < 0.001; hippocampus: *t* = −4.01, df = 3184, *p* < 0.001; lOFC: *t* = −4.59, df = 3184, *p* < 0.001; mOFC: *t* = −4.56, df = 3184, *p* < 0.001). Mothers of participants with missing data were younger at the time of inclusion (*t* = −9.8, df = 576.91, *p* < 0.001), were less educated (*Χ* = 143.39, df = 2, *p* < 0.001) and more often had a non‐Western background (*Χ* = 164.63, df = 2, *p* < 0.001). There were no differences in the age and sex of the child.

### 3.2 Population Characteristics

The characteristics of the population are shown in Table 1. Our sample consisted of 2729 participants, of whom 50.3% were female. Compared to boys, girls had a lower BMI‐SDS, showed more RE, and had smaller brain volumes.

### 3.3 BMI‐SDS and Brain Volumes in Girls

**FIGURE 2:**
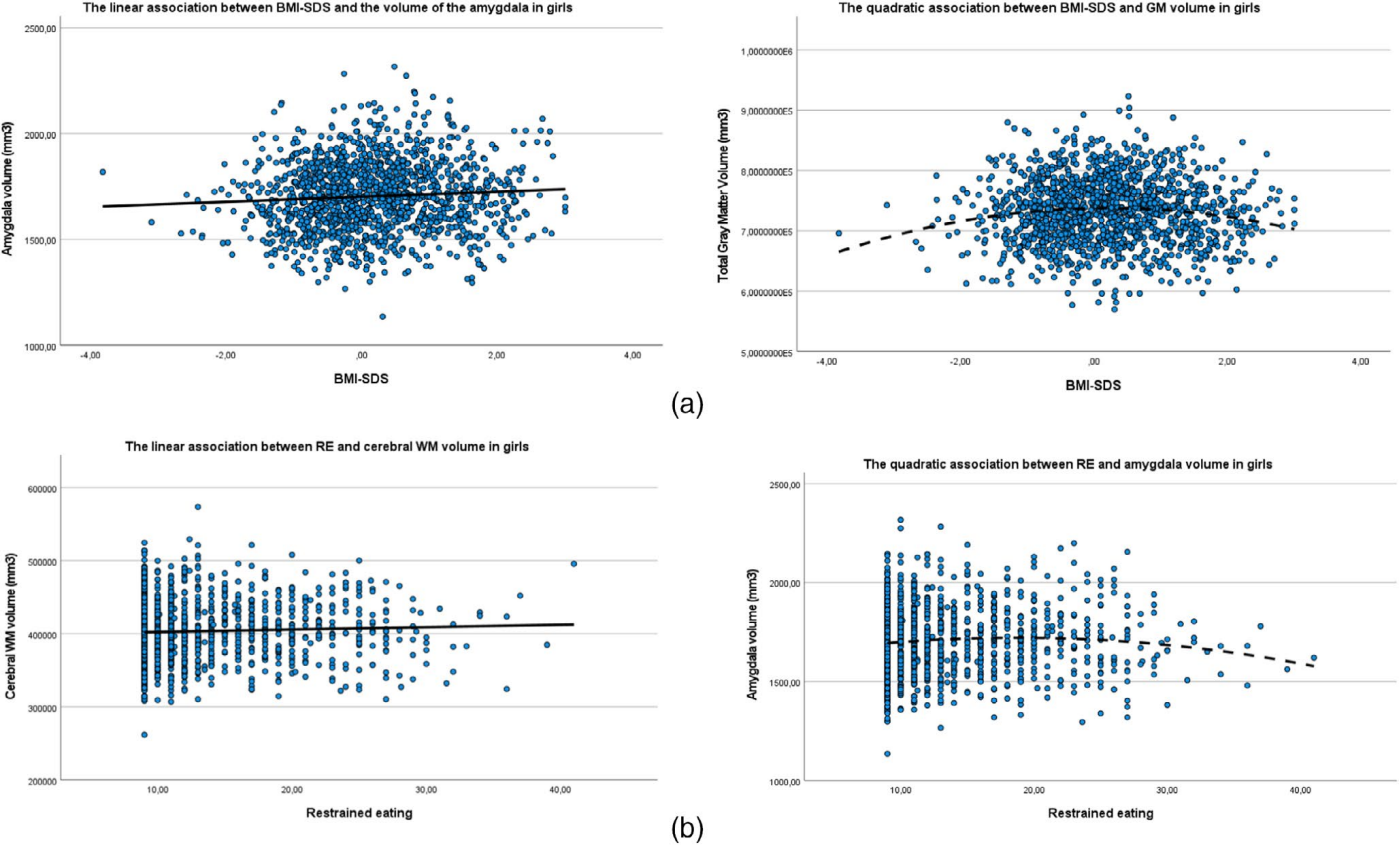
(a) Associations between BMI‐SDS and different brain volumes in girls and (b) associations between restrained eating and different brain volumes in girls. BMI‐SDS = body mass index‐standard deviation score; GM = gray matter volume; RE = restrained eating; WM = white matter volume.

The results presented in Table 2 demonstrated positive linear associations of BMI‐SDS with TBV, amygdala, cingulate, hippocampus, lOFC, mOFC, and insula, meaning that a higher BMI‐SDS in girls was associated with larger volumes of these brain structures (see Figure 2). The associations between BMI‐SDS and total GM and WM volumes were quadratic, indicating that the girls with the lowest and highest BMI‐SDS had the smallest brain volumes (see Figure 2). These analyses were adjusted for covariates and remained significant after correction for multiple testing.

**TABLE 2:** The association between BMI‐SDS and brain volumes in girls.

Outcome	Model	*B*	*p* ^b^	95% CI for *B*		*R* ^2^ change BMI
				Lower	Upper	
Total intracranial volume (mm^3^)	Linear	7345.56	0.021^b^	1131.35	13,559.78	0.004
Total gray matter volume (mm^3^)	Quadratic	−2619.29	0.011^b^	−4634.33	−604.24	0.005
Total cerebral white matter volume (mm^3^)	Quadratic	−2091.47	0.009^b^	−3670.75	−512.18	0.007
Amygdala (mm^3^)^a^	Linear	14.72	< 0.001^b^	6.96	22.48	0.012
Cingulate (mm^3^)^a^	Linear	108.06	< 0.001^b^	55.51	160.62	0.005
Hippocampus (mm^3^)^a^	Linear	18.02	0.019^b^	2.94	33.10	0.003
Lateral orbitofrontal cortex (mm^3^)^a^	Linear	74.43	< 0.001^b^	33.28	116.17	0.002
Medial orbitofrontal cortex (mm^3^)^a^	Linear	49.27	0.001^b^	19.38	79.15	0.002
Insula (mm^3^)^a^	Linear	50.04	0.003^b^	16.54	83.54	0.004

*Note:* Analyses are adjusted for age and ethnicity of the child and age, educational level, and alcohol consumption of the mother.

Abbreviation: *B* = unstandardized beta.

^a^ Analyses are also adjusted for total intracranial volume.

^b^ Analyses remain significant after Benjamini–Hochberg correction. Curve fitting results of all models are presented in Table S3.

For all significant results described, the overall effect sizes were very small, ranging from *R* ^2^ = 0.002–0.012. Sensitivity analyses in which BMI‐SDS was divided into subgroups confirmed results for most brain structures (see Table S1).

### 3.4 RE and Brain Volumes in Girls

Table 3 shows that RE was linearly associated with greater GM and WM volumes, and volumes of the hippocampus and mOFC. We also found a negative quadratic association between RE and amygdala and insula volumes, indicating that girls with the least and the most RE had the smallest amygdala and insula volumes. These associations remained significant after correction for multiple testing, except for the analyses with total GM as the outcome. See Figure 2b for a visual overview of the different association curves. When additionally adjusting the significant associations for BMI‐SDS (Table 4), all associations remained significant, except for the association with the mOFC and the insula. Again, effect sizes were very small and ranged from *R* ^2^ = 0.001–0.013.

**TABLE 3:** The association between restrained eating and brain volumes in girls.

Outcome	Model	*B*	*p* ^b^	95% CI for *B*		*R* ^2^ change RE
				Lower	Upper	
Total intracranial volume (mm^3^)	Linear	717.29	0.220	−428.29	1862.87	0.000
Total gray matter volume (mm^3^)	Linear	536.88	0.045	10.93	1062.83	0.002
Total cerebral white matter volume (mm^3^)	Linear	625.18	0.003^b^	215.94	1034.42	0.004
Amygdala (mm^3^)^a^	Quadratic	−0.26	0.007^b^	−0.45	−0.07	0.003
Cingulate (mm^3^)^a^	Linear	9.56	0.052	−0.07	19.20	0.000
Hippocampus (mm^3^)^a^	Linear	3.93	0.005^b^	1.18	6.68	0.003
Lateral orbitofrontal cortex (mm^3^)^a^	Linear	6.05	0.118	−1.54	13.63	0.000
Medial orbitofrontal cortex (mm^3^)^a^	Linear	7.05	0.011^b^	1.59	12.52	0.001
Insula (mm^3^)^a^	Quadratic	−0.95	0.024^b^	−1.77	−0.12	0.001

*Note:* Analyses are adjusted for age and ethnicity of the child and age, educational level, and alcohol consumption of the mother during pregnancy.

Abbreviations: *B* = unstandardized beta; RE = restrained eating.

^a^ Analyses are also adjusted for total intracranial volume.

^b^ Analyses remain significant after Benjamini–Hochberg correction. Curve fitting results of all models are presented in Table S3.

**TABLE 4:** The association between restrained eating and brain volumes in girls, additionally corrected for BMI‐SDS.

Outcome	Model	*B*	*p* ^b^	95% CI for *B*		*R* ^2^ change RE
				Lower	Upper	
Total cerebral white matter volume (mm^3^)	Linear	519.42	0.025	65.67	973.16	0.013
Amygdala (mm^3^)^a^	Quadratic	−0.23	0.018	−0.42	−0.04	0.009
Hippocampus (mm^3^)^a^	Linear	3.09	0.045	0.06	6.12	0.001
Medial orbitofrontal cortex (mm^3^)^a^	Linear	3.97	0.196	−2.04	9.98	0.001
Insula (mm^3^)^a^	Quadratic	−2.42	0.488	−9.24	4.41	0.004

*Note:* Only significant associations from Table 3 are used for additional correction with BMI‐SDS. Analyses are adjusted for age and ethnicity of the child and age, educational level, and alcohol consumption of the mother during pregnancy. Curve fitting results of all models are presented in Table S3.

Abbreviations: *B* = unstandardized beta; RE = restrained eating.

^a^ Analyses are also adjusted for total intracranial volume.

^b^ Analyses remain significant after Benjamini–Hochberg correction.

The results of the sensitivity analyses in which RE is divided into subgroups confirmed the results of the main analysis with the amygdala as outcome (Table S5), while the other brain metrics were no longer statistically significant.

### 3.5 Associations Between BMI‐SDS, RE, and Brain Volumes in Boys

The results of the analyses with BMI‐SDS as a predictor of brain volumes in boys are shown in Table S6. We found significant positive linear associations between BMI‐SDS and most total and local brain volumes. For the association between BMI‐SDS and TBV, the quadratic model had the best fit. In contrast with the girls, the association between BMI‐SDS and the lOFC was not significant in boys. All associations survived correction for multiple testing. The overall effect sizes for the significant results were very small (*R* ^2^ = 0.001–0.013). Sensitivity analyses in which BMI‐SDS was divided into subgroups mostly confirmed our main findings (see Table S7).

None of the associations of RE with the different brain volumes were significant in boys (see Table S8), and therefore, no further analyses (additional adjustment for BMI‐SDS, and analyses in subgroups of RE) were done.

## 4 Discussion

This study is unique in examining the relationship between RE, BMI‐SDS, and brain volumes in a pediatric population‐based birth cohort in which sex differences and the shape of the association are studied. Our results suggest that BMI‐SDS and RE are both significantly associated with a wide range of brain volumes in girls, although the shape of the associations differed between brain structures (i.e., BMI‐SDS is positive linear associated with TBV, amygdala, cingulate, hippocampus, lOFC, mOFC and insula and negative quadratic associated with total GM and total WM). In girls, the association between RE and the amygdala(negatively quadratic) remains significant after correction for multiple testing and after adjustment for BMI‐SDS. This outcome is in contrast to the association between RE and total WM, hippocampus, mOFC volume, and insula (resp. positively linear and negative quadratic, which remain significant after correction for multiple testing, but not after adjusting for BMI‐SDS). In exploratory analysis, we analyzed the same associations in boys. As in girls, we find significant associations between BMI‐SDS and a wide range of brain volumes, but most of the associations show a positive linear association with total and local brain volumes. In boys, most associations do not survive correction for multiple testing, suggesting these findings are not equally strong. Interestingly, RE is not associated with any of the investigated brain volumes in boys as opposed to girls.

### 4.1 BMI‐SDS and Brain Volumes

Our findings are in line with those of the ABCD study, which is the most comparable to our population, both in terms of number and age of the children and brain structures (Bohon and Welch 2021). In the ABCD study, an inverted U‐shaped association between BMI‐SDS and total GM volume is also found. In addition, our findings align with clinical studies in adolescents with AN (Kappou et al. 2021; Seitz et al. 2014, 2015, 2016; Herpertz‐Dahlmann et al. 2011; Bernardoni et al. 2018), who generally have a severely low BMI‐SDS and show decreased GM volumes. Effect sizes in those studies are small, like in ours, suggesting that multiple factors underlie the association between BMI‐SDS and brain volumes.

Our results differ from those of Kennedy et al. (2016) and Alosco et al. (2014), who performed their studies in samples of individuals with a mean age of 13 and 14 years old, respectively. We find mostly positive linear associations between BMI‐SDS and a range of brain volumes, whereas these studies find negative linear associations in which a higher BMI‐SDS is associated with decreased brain volumes. Several explanations for the different results can be considered. First, Kennedy et al. (2016) and Alosco et al. (2014) do not examine nonlinearity in their data, which raises questions about the exact shape of the association in their studies and whether this differs from our findings. Second, the variance in our results can potentially be attributed to our notably larger sample size in contrast to the studies of Kennedy et al. (2016) (*n* = 137) and Alosco et al. (2014) (*n* = 120). Third, our study examines sex differences, whereas the studies of Alosco et al. (2014) and Kennedy et al. (2016) combine boys and girls without considering potential sex‐related diverging effects. Fourth, our non‐response analysis shows that our sample has a relatively large amount of missing data from participants with a higher BMI‐SDS and smaller brain volumes, which can influence the shape of the association found. In addition, the likelihood of selection bias in our results cannot be excluded. However, as Alosco et al. (2014) and Kennedy et al. (2016) do not discuss non‐response analyses in their studies, there is no information on possible selection bias in their results. Finally, during adolescence, the typically developing brain undergoes major changes (Lenroot and Giedd 2006) and age is one of the factors that greatly influences GM volumes, in which, beginning in adolescence, an increasing age is associated with decreasing GM volumes. The present study is characterized by a highly restricted age range, with data collection preceding any age‐related decline in TBV. It is plausible that non‐linear effects of age are negligible within the present study's context. However, in the studies conducted by Alosco et al. (2014) and Kennedy et al. (2016), this process has likely already commenced and may have exerted an additional deleterious effect on GM volumes.

The present findings demonstrate that analogous cerebral structures appear to be associated with BMI in children with a normal range BMI as in children and adults with extremes of body weight, for example, obesity and AN. However, it remains unclear whether these differences in brain morphology should be regarded as biomarkers or as part of pathophysiological mechanisms due to the lack of longitudinal studies. Several hypotheses can be considered.

First, it can be hypothesized that children with lower or higher BMI‐SDS might adjust their behavior in an attempt to maintain a healthy body weight. An increase in specific eating behaviors can, for example, lead to synaptogenesis and an increase in related GM brain structures (Draganski et al. 2004; Taubert et al. 2010). Second, genetic factors interact with environmental factors, including parental eating patterns and parental feeding practices, and this interaction might shape the BMI‐SDS status of the child (Jansen et al. 2012, 2017; Scaglioni et al. 2008). Another argument supporting the idea that genetic factors play a role in BMI‐SDS status is that people from different ethnic backgrounds appear to be differentially susceptible to becoming overweight in comparable environments (Ogden et al. 2006; Kral and Faith 2009). Third, other factors, including psychopathology, can have a mediating role in the association between BMI‐SDS and brain morphology. For example, literature shows that depressive symptoms and anxiety in children are associated with a lower BMI‐SDS and changes in brain morphology (Bohon and Welch 2021; Burke and Storch 2015). Indeed, considering the small effect sizes of these associations, other factors likely underlie the association between BMI‐SDS and brain morphology. Future research regarding eating disorder‐related features in the general pediatric population should therefore be focused on associations with brain morphology, but also incorporate other factors that are associated with eating disorders, including comorbid psychopathology, (neuro)psychological functioning, family dynamics, and personality traits.

### 4.2 RE and Brain Volumes in Girls

Interestingly, our findings demonstrate that smaller volumes of the amygdala and insula are associated with less and more RE. The amygdala is involved in processing emotions, especially fear, and is part of the limbic system. The insula subserves a wide variety of functions in the limbic system; for example, its central role in integrating information about the emotional state of the body into a cognitive representational map of the resultant feeling state (Damasio 1996; Uddin et al. 2017). Nunn et al. (2011) hypothesize that insula dysfunction, specifically disconnection, may underlie many of the features of AN. Our findings may support this hypothesis, since more and less RE are associated with smaller volumes of the insula and might thus be already present in the healthy population. Due to the cross‐sectional character of our study, differentiation between brain volumes as biomarkers versus pathophysiological factors is limited. More longitudinal studies need to be conducted to address this.

Contrary to our initial hypothesis, a greater degree of RE is found to be associated with larger volumes of the medial temporal lobe, the hippocampus, and medial OFC volumes. Several explanations can be considered to account for this finding. First, our initial hypothesis is based on the literature concerning individuals in the acute phase of AN, who exhibit extreme RE, extremely low BMI‐SDS, and reduced brain volumes. Various explanations can be considered. Changes in brain structures in AN mostly result from starvation. Another explanation is that individuals with AN often restrict high‐caloric foods that contain essential polyunsaturated fatty acids, proteins, and nutrients (Schebendach et al. 2019; Coniglio et al. 2017; Mayer et al. 2012). These nutrients are important for brain function, and deficiency is associated with a disruption of normal cortical maturation during adolescence (Galler et al. 2017). It can be hypothesized that the extent to which children from the Generation R population restrict their diets and consequently experience nutrient deficiencies is much less severe or absent in comparison with individuals with AN. As a consequence, the brains of the children in the Generation R Study are therefore much less affected.

A secondary rationale for the discrepancy between our hypothesis and the identified association lies in the limitations of the total score on the Restrained Eating scale of the DEBQ. This score struggles to distinguish between attempts to restrain eating and actual eating behavior. As a result, the DEBQ may not fully capture the actual dietary restriction seen in restrictive eating disorders such as AN. Moreover, in children and adolescents, RE often alternates with periods of food approach behavior and overeating. Frequent patterns of these alternating eating behaviors are considered disordered eating behavior (Schuck et al. 2018; Herpertz‐Dahlmann et al. 2008; Croll et al. 2002) and in some cases are linked to a higher BMI (Larsen et al. 2007). Interestingly, food approach behavior itself is associated with higher GM and WM volumes in the Generation R population (Kennedy et al. 2016; Dmitrichenko et al. 2022). Although food approach behavior could potentially confound the relationship between RE and brain volume, it is more likely to act as a mediator or antecedent in the causal pathway. This is why we chose not to adjust for it in our analysis. In conclusion, further examination of RE in different forms (actual versus intentional RE) and associated eating behaviors, in relation to brain morphology and in relation to BMI‐SDS, may offer valuable insights into the observed associations between RE and brain structures.

### 4.3 Strengths and Limitations

This study has a number of methodological strengths. First, as part of the larger Generation R cohort, this study has a longitudinal, prospective, population‐based design with a large number of participants. Because participants are enrolled prenatally, the likelihood of selection bias is reduced.

A second strength is the ability to adjust for multiple covariates, which increases the reliability of our findings. Third, we examined which model best fitted the association between eating disorder‐related traits and brain volumes, allowing us to examine the relationships between BMI‐SDS, RE, and brain morphology in more detail. Fourth, due to the large sample size, we are able to stratify our analyses between typically developing girls and boys. Our study shows that it is important to study these groups separately because our results are different, suggesting that the underlying mechanism for associations between BMI‐SDS, RE, and brain morphology might be different for boys and girls.

A number of limitations should also be noted. First, information on RE is collected from parents (mostly the mothers). There is a possibility that the parent may report that the child is restraining his/her diet when in fact the child is not, leading to erroneous information. It is also possible that the child is restraining their eating, or at least trying to, but the parent is not aware. However, a validation study of the Child Eating Behavior Questionnaire (CEBQ), a questionnaire used to assess a wide range of appetitive traits in children, finds a moderate association between children's behavioral measures of eating and parent‐reported CEBQ. This supports that mothers can indeed accurately report on their child's eating behavior. Second, almost half of the participants' mothers in our population have a high economic status, whereas only 4.4% of the participants' mothers report a low educational level. This may affect the generalizability of findings. Third, restrictive eating behavior, as seen in individuals with AN, has been operationalized as RE in this study. While both RE and restrictive eating involve controlling food intake, the constructs differ in that RE captures the intention and attempt to control eating, whereas restrictive eating captures the actual controlled/restricted eating behavior (Herman and Polivy 1975; Herman and Mack 1975). Longitudinal studies would be valuable to explore whether RE might serve as a precursor or predictor of eating disorders, including AN. In these studies, it would be valuable to look at both the attempt to control eating and the actual restrictive eating behavior, which would provide a more comprehensive understanding of eating behavior as seen in eating disorders, specifically AN. Fourth, in this study, non‐response was related to important demographic and clinical characteristics, including BMI, brain volume, ethnicity, and parental education. This may have biased certain results, although multiple imputation is performed for missing values of these variables, reducing the likelihood of bias. Finally, due to our cross‐sectional design, we cannot infer causality of our findings. Therefore, it is important to follow these children over time to investigate whether the associations found in middle childhood are also present during adolescence and young adulthood.

### 4.4 Conclusions and Future Directions

This study provides evidence for associations between eating disorder related characteristics (i.e., BMI‐SDS and RE) and both global and local brain structures in 9‐year‐old girls from the general population. Also, in boys, BMI‐SDS is associated with a broad range of brain volumes, but the shape of the associations differs from most of the associations found in girls. Our findings add important information to the current knowledge of the associations between RE and brain morphology during childhood, before the common age of onset of AN.

Furthermore, future research should examine a wider range of RE in terms of severity and type. Conducting such research will augment our comprehension of the fundamental risk elements and antecedents linked to the development of eating disorders.

## Author Contributions

**M. E. J. Deen:** formal analysis, writing – original draft. **C. P. M. Steegers:** formal analysis, writing – original draft. **P. W. Jansen:** writing – review and editing. **T. White:** writing – review and editing. **K. F. M. Bracké:** writing – review and editing. **M. H. J. Hillegers:** writing – review and editing. **G. C. Dieleman:** writing – original draft, writing – review and editing.

## Conflicts of Interest

The authors declare no conflicts of interest.

## Supporting information


**Data S1.** Tables.

## Data Availability

The datasets analyzed in the current study are not publicly available due to privacy restrictions. Data from the Generation R Study are available upon reasonable request (generationr@erasmusmc.nl), subject to local rules and regulations.
