# Supplementary material for: Restrained Eating Features and Brain Morphology: A Pediatric Population‐Based Study
Source: Int J Eat Disord. 2025 Apr 26;58(8):1440–51. doi: 10.1002/eat.24445 (PMC12336774; doi:10.1002/eat.24445)
Supplement: Supplementary file 1 — Data S1. Tables. [file EAT-58-1440-s001.docx]

SUPPLEMENTARY TABLES

| Supplementary table 1. Correlation matrix for girls | | | | | | | | | | | | | | | | |
| --- | --- | --- | --- | --- | --- | --- | --- | --- | --- | --- | --- | --- | --- | --- | --- | --- |
|  | **Age** | **BMI-SDS** | **Restrained eating** | **Age of the mother** | **Education of the mother** | **Ethnicity** | **Maternal alcohol use during pregnancy** | **Total int. volume (mm^3^)** | **Total GM volume (mm^3^)** | **Total WM volume (mm^3^)** | **Amygdala (mm^3^)** | **Cingulate (mm^3^)** | **Hippo-campus (mm^3^)** | **lOFC (mm^3^)** | **mOFC (mm^3^)** | **Insula**  **(mm^3^)** |
| Age | 1 | .10** | .11** | -.13** | -.14** | .14** | -.04 | -.05 | -.08** | -.02 | -.03 | -.07* | -.03 | -.07* | -.02 | -.05 |
| BMI-SDS |  | 1 | .46** | -.14** | -.22** | .21** | -.15** | -.01 | -.01 | .04 | .07** | .06* | .04 | .05 | .07** | .04 |
| Restrained eating score |  |  | 1 | -.08** | -.17** | .19** | -.11** | -.02 | -.02 | .04 | .02 | .02 | .05 | .01 | .05 | -.014 |
| Age of the mother |  |  |  | 1 | .34** | -.19** | .25** | .10** | .14** | .06* | .07** | .09** | .09** | .11** | .10** | .10** |
| Education of the mother |  |  |  |  | 1 | -.31** | .26** | .19** | .22** | .12** | .10** | .14** | .12** | .15** | .09** | .17** |
| Ethnicity † |  |  |  |  |  | 1 | -.25** | -.19** | -.25** | -.14** | -.11** | -.14** | -.10** | -.17** | -.09** | -.14** |
| Maternal alcohol use during pregnancy †† |  |  |  |  |  |  | 1 | .13** | .13** | .06* | .09** | .10** | .08 | .05 | .04 | .07* |
| Total int. volume (mm^3^) |  |  |  |  |  |  |  | 1 | .87** | .85** | .52** | .71** | .56** | .64** | .61** | .62** |
| Total GM volume (mm^3^) |  |  |  |  |  |  |  |  | 1 | .78** | .60** | .79** | .59** | .75** | .68** | .73** |
| Total WM volume (mm^3^) |  |  |  |  |  |  |  |  |  | 1 | .54** | .63** | .55** | .60** | .57** | .54** |
| Amygdala (mm^3^) |  |  |  |  |  |  |  |  |  |  | 1 | .49** | .63** | .46** | .42** | .43** |
| Cingulate (mm^3^) |  |  |  |  |  |  |  |  |  |  |  | 1 | .48** | .62** | .59** | .61** |
| Hippocampus (mm^3^) |  |  |  |  |  |  |  |  |  |  |  |  | 1 | .44** | .40** | .44** |
| lOFC(mm^3^) |  |  |  |  |  |  |  |  |  |  |  |  |  | 1 | .66** | .62** |
| mOFC (mm^3^) |  |  |  |  |  |  |  |  |  |  |  |  |  |  | 1 | .51** |
| Insula (mm^3^) |  |  |  |  |  |  |  |  |  |  |  |  |  |  |  | 1 |
| ** *Correlation is significant at 0.01 level (2-tailed). * Correlation is significant at 0.05 level (2-tailed); BMI-SDS=Body Mass Index-Standard Deviation Score; Total int. volume=total intracranial volume; lOFC=lateral orbitofrontal cortex; mOFC= medial orbitofrontal cortex, . ^#^ 1=low maternal educational level, 2=medium maternal educational level, 3=high maternal educational level (also see table 1), † 1=Dutch nationality, 2=other Western nationality, 3= non-Western nationality, †† 0= no maternal alcohol usage during pregnancy, 1= maternal alcohol usage until pregnancy, 2=occasional maternal alcohol usage during pregnancy, 3=frequent maternal alcohol usage during pregnancy* | | | | | | | | | | | | | | | | |

| Supplementary table 2. Correlation matrix for boys | | | | | | | | | | | | | | | | |
| --- | --- | --- | --- | --- | --- | --- | --- | --- | --- | --- | --- | --- | --- | --- | --- | --- |
|  | **Age** | **BMI-SDS** | **Restrained eating** | **Age of the mother** | **Education of the mother** | **Ethnicity** | **Maternal alcohol use during pregnancy** | **Total int. volume (mm^3^)** | **Total GM volume (mm^3^)** | **Total WM volume (mm^3^)** | **Amygdala (mm^3^)** | **Cingulate (mm^3^)** | **Hippo-campus (mm^3^)** | **lOFC (mm^3^)** | **mOFC (mm^3^)** | **Insula**  **(mm3)** |
| Age | 1 | .08** | .06* | -.11** | -.07* | .14*** | -.01 | .01 | -.03 | .03 | .01 | -.00 | .03 | -.01 | -.06* | .00 |
| BMI-SDS |  | 1 | .42** | -.09** | -.21** | .18** | -.13** | -.03 | .00 | .05 | .09** | .06* | .06* | .01 | .03 | .03 |
| Restrainedeating score |  |  | 1 | -.03 | -.10** | .20** | -.11** | -.08*** | -.06* | -.01 | -.01 | -.03 | -.04 | -.03 | -.04 | -.05 |
| Age of the mother |  |  |  | 1 | .27** | -.21** | .23** | .13** | .13** | .09** | .05 | .10** | .04 | .08** | .06* | .06* |
| Education of the mother^#^ |  |  |  |  | 1 | -.33** | .32** | .1** | .19** | .12** | .08** | .09** | .09** | .11** | .10** | .11** |
| Ethnicity† |  |  |  |  |  | 1 | -.31** | -.25** | -.26** | -.18** | -.13** | -.15** | -.14** | -.16** | -.16** | -.16** |
| Maternal alcohol use during pregnancy†† |  |  |  |  |  |  | 1 | .19** | .18** | .12** | .07* | .13** | .10** | .11** | .14** | .12** |
| Total int. volume (mm^3^) |  |  |  |  |  |  |  | 1 | .87** | .86** | .52** | .70** | .53** | .61** | .61** | .62** |
| Total GM volume (mm^3^) |  |  |  |  |  |  |  |  | 1 | .79** | .60** | .80** | .57** | .73** | .68** | .71** |
| Total WM volume (mm^3^) |  |  |  |  |  |  |  |  |  | 1 | .54** | .64** | .52** | .58** | .57** | .55** |
| Amygdala (mm^3^) |  |  |  |  |  |  |  |  |  |  | 1 | .48** | .62** | .41** | .43** | .44** |
| Cingulate (mm^3^) |  |  |  |  |  |  |  |  |  |  |  | 1 | .49** | .62** | .58** | .61** |
| Hippocampus (mm^3^) |  |  |  |  |  |  |  |  |  |  |  |  | 1 | .40** | .36** | .44** |
| lOFC(mm^3^) |  |  |  |  |  |  |  |  |  |  |  |  |  | 1 | .67** | .60** |
| mOFC (mm^3^) |  |  |  |  |  |  |  |  |  |  |  |  |  |  | 1 | .51** |
| Insula (mm^3^) |  |  |  |  |  |  |  |  |  |  |  |  |  |  |  | 1 |
| ** Correlation is significant at 0.01 level (2-tailed). * Correlation is significant at 0.05 level (2-tailed); BMI-SDS=Body Mass Index-Standard Deviation Score; Total int. volume=total intracranial volume; lOFC=lateral orbitofrontal cortex; mOFC= medial orbitofrontal cortex, *^#^ 1=low maternal educational level, 2=medium maternal educational level, 3=high maternal educational level (also see table 1), † 1=Dutch nationality, 2=other Western nationality, 3= non-Western nationality, †† 0= no maternal alcohol usage during pregnancy, 1= maternal alcohol usage until pregnancy, 2=occasional maternal alcohol usage during pregnancy, 3=frequent maternal alcohol usage during pregnancy* | | | | | | | | | | | | | | | | |

| Supplementary table 3: Curve fitting analyses | | | | | | | | | | |
| --- | --- | --- | --- | --- | --- | --- | --- | --- | --- | --- |
| BMI girls | | | | | | | | | | |
|  | | **Total Int.** | **Total GM** | **Total WM** | **Amygdala** | **Cingulate** | **Hippocampus** | **Lateral OFC** | **Medial OFC** | **Insula** |
| Linear | R^2^ | .062 | .091 | .032 | .272 | .489 | .309 | .411 | .002 | .481 |
|  | P (full model) | <.001 | <.001 | <.001 | <.001 | <.001 | <.001 | <.001 | <.001 | .000 |
|  | B (variable) | 7345.564 | 4333.927 | 3824.573 | 14.717 | 108.063 | 18.019 | 74.729 | 49.265 | 48.74 |
|  | R2 change | .004 | .007 | .008 | .012 | .005 | .003 | .002 | .002 | .003 |
|  | P (change) | .021 | .003 | <.001 | <.001 | 0.001 | .019 | <.001 | .001 | <.001 |
| Quadratic | R^2^ | .065 | .096 | .039 | .273 | .489 | .310 | .412 | .37 | .484 |
|  | P (full model) | <.001 | <.001 | <.001 | <.001 | <.001 | <.001 | <.001 | <.001 | .000 |
|  | B (variable) | -3906.920 | -2619.286 | -2091.466 | -1.258 | -1.632 | -5.410 | -19.678 | -9.967 | -16.11 |
|  | R^2^ change | .003 | .005 | .007 | .000 | .000 | .001 | .001 | .000 | .001 |
|  | P (change) | .082 | .011 | .009 | .654 | .932 | .323 | .190 | .358 | .100 |
| Restrained eating girls | | | | | | | | | | |
|  | | **Total Int.** | **Total GM** | **Total WM** | **Amygdala** | **Cingulate** | **Hippocampus** | **Lateral OFC** | **Medial OFC** | **Insula** |
| Linear | R^2^ | .058 | .086 | .028 | .263 | .485 | .309 | .409 | .365 | .402 |
|  | P (full model) | <.001 | <.001 | <.001 | <.001 | <.001 | <.001 | <.001 | <.001 | <.001 |
|  | B (variable) | 717.292 | 536.879 | 625.182 | 1.205 | 9.563 | 3.929 | 6.046 | 7.054 | 1.397 |
|  | R^2^ change | .000 | .002 | .004 | .002 | .000 | .003 | .000 | .001 | .001 |
|  | P (change) | .220 | .045 | .003 | .095 | .052 | .005 | .118 | .011 | .655 |
| Quadratic | R^2^ | .058 | .087 | .032 | .265 | .485 | .309 | .409 | .365 | .403 |
|  | P (full model) | <.001 | <.001 | <.001 | <.001 | <.001 | <.001 | <.001 | <.001 | <.001 |
|  | B (variable) | -17.045 | -32.528 | -38.525 | -.261 | -.407 | -.011 | -.228 | -.214 | -.944 |
|  | R^2^ change | .001 | .001 | .003 | .003 | .000 | .000 | .000 | .000 | .001 |
|  | P (change) | .827 | .364 | .168 | .007 | .538 | .954 | .662 | .567 | .024 |
| BMI boys | | | | | | | | | | |
|  |  | **Total Int.** | **Total GM** | **Total WM** | **Amygdala** | **Cingulate** | **Hippocampus** | **Lateral OFC** | **Medial OFC** | **Insula** |
| Linear | R^2^ | .094 | .091 | .058 | .289 | .469 | 0286 | .360 | .364 | .383 |
|  | P (full model) | <.001 | <.001 | <.001 | <.001 | <.001 | <.001 | <.001 | <.001 | <.001 |
|  | B (variable) | 5103.176 | 4091.587 | 4189.98 | 18.757 | 104.007 | 25.22 | 32.866 | 39.939 | 44.237 |
|  | R^2^ change | .003 | .007 | .013 | .009 | .006 | .006 | .001 | .002 | .003 |
|  | P (change) | .138 | .008 | <.001 | <.001 | <.001 | .003 | .158 | .017 | .021 |
| Quadratic | R^2^ | .097 | .094 | .059 | .290 | .469 | .286 | .360 | .364 | .383 |
|  | P (full model) | <.001 | <.001 | <.001 | <.001 | <.001 | <.001 | <.001 | <.001 | <.001 |
|  | B (variable) | -6997.216 | -3023.942 | -1822.89 | 3.137 | -8.804 | -.31 | -2.408 | 8.584 | -12.956 |
|  | R^2^ change | .003 | .003 | .001 | .000 | .000 | .000 | .000 | .000 | .001 |
|  | P (change) | .004 | .005 | .037 | .313 | .674 | .959 | .883 | .466 | .337 |
| Restrained eating boys | | | | | | | | | | |
|  |  | **Total Int.** | **Total GM** | **Total WM** | **Amygdala** | **Cingulate** | **Hippocampus** | **Lateral OFC** | **Medial OFC** | **Insula** |
| Linear | R^2^ | .092 | .084 | 0.047 | .281 | .464 | .281 | .358 | .362 | .379 |
|  | P (full model) | <.001 | <.001 | <0.001 | <.001 | <.001 | <.001 | <.001 | <.001 | <.001 |
|  | B (variable) | -512.204 | -15.757 | 234.46 | 1.166 | 5.175 | .002 | 2.697 | 1.706 | -.571 |
|  | R^2^ change | .000 | .000 | .001 | .002 | .000 | .000 | .000 | .000 | .000 |
|  | P (change) | .454 | .959 | .344 | .187 | .384 | .999 | .561 | .609 | .881 |
| Quadratic | R^2^ | .093 | .085 | .048 | .283 | .464 | .281 | .358 | .363 | .380 |
|  | P (full model) | <.001 | <.001 | <.001 | <.001 | <.001 | <.001 | <.001 | <.001 | <.001 |
|  | B (variable) | -111.061 | -59.624 | -72.08 | -.239 | .617 | -.169 | -.017 | -.684 | -.062 |
|  | R^2^ change | .001 | .001 | .001 | .001 | .000 | .000 | .000 | .001 | .000 |
|  | P (change) | .321 | .237 | .075 | .099 | .526 | .544 | .982 | .209 | .921 |
| *For P (full model), the mean is taken from 5 original data sets. Total int. = Total intracranial volume; Total GM=Total Gray Matter volume; Total WM=Total White Matter volume; Lateral OFC=Lateral Orbitofrontal Cortex; Medial OFC=Medial Orbitofrontal Cortex; R^2^ change indicates the change between models (covariates only vs. covariates plus linear BMI vs. covariates plus linear and quadratic BMI; P (change) refers to means the p value from model comparison (linear vs. covariates-only model, and quadratic vs. linear model)* | | | | | | | | | | |

| Supplementary table 4A. The association between BMI-SDS subgroups^#^ and brain volumes in girls for linear models. | | | | | | | | | |
| --- | --- | --- | --- | --- | --- | --- | --- | --- | --- |
|  | **Split** | **BMI-SDS subgroups** | | | | **df** | **F** | **η_p_^2^** | **P-value** |
|  |  | **Lower group** | | **Upper group** | |  |  |  |  |
| Outcome |  | **N** | **M(SD)** | **N** | **M(SD)** |  |  |  |  |
| Total intracranial volume (mm^3^) | 20/80 | 233 | 1.43*10^6^ (1.02*10^5^) | 906 | 1.45*10^6^ (1.16*10^5^) | 1 | 10.98 | .010 | <.001 |
| Amygdala (mm^3^)† | 20/80 | 233 | 1666.89 (149.05) | 906 | 1716.12 (165.61) | 1 | 12.42 | .011 | <.001 |
| Cingulate (mm^3^) † | 20/80 | 233 | 11834.37 (1256.41) | 906 | 12188.75 (1331.00). | 1 | 8.97 | .008 | .003 |
| Hippocampus (mm^3^)† | 20/80 | 233 | 3865.52 (309.68) | 906 | 3933.01 (320.59) | 1 | 4.101 | .004 | .043 |
| Lateral orbitofrontal cortex (mm^3^)† | 20/80 | 233 | 9082.04 (948.05) | 906 | 9241.19 (990.75) | 1 | 1.140 | .001 | .235 |
| Medial orbitofrontal cortex (mm^3^)† | 20/80 | 233 | 6109.11 (655.92) | 906 | 6252.60 (682.11) | 1 | 2.82 | .002 | .093 |
| Insula (mm^3^) | 20/80 | 233 | 7694.16 (782.70) | 906 | 7847.33 (777.33) | 1 | 3.69 | .003 | .055 |
| *Note: ^#^BMI-SDS is split by the lowest 20% versus the highest 80%. Analyses are adjusted for age and ethnicity of the child and age, educational level and alcohol use of the mother during pregnancy. †Analyses are also adjusted for total intracranial volume. Effect estimates are based on ANCOVA’s with F=F-test of significance, df=degrees of freedom and η_p_^2^=partial eta-squared .* | | | | | | | | | |

| **Supplementary table 4B. Complementary analysis of the linear relationship between BMI-SDS and brain volumes in subgroups of girls, supplementing quadratic models.** | | | | | | | |
| --- | --- | --- | --- | --- | --- | --- | --- |
| **Outcome** | **BMI-SDS Subgroup** | **N** | **B** | **P-value** | **95% CI for B** | | **R^2^ change BMI-SDS** |
|  |  |  |  |  | **Lower** | **Upper** |  |
| Total grey matter volume (mm^3^) | Low | 582 | 868.719 | .814 | -6382.886 | 8120.324 | .000 |
|  | High | 559 | 1660.289 | .697 | -6702.676 | 10023.254 | .000 |
| Total cerebral white matter volume (mm^3^) | Low | 582 | 215.701 | .940 | -5442.702 | 5874.103 | .002 |
|  | High | 559 | -887.297 | .792 | -7490.546 | 5715.953 | .000 |
| Cingulate (mm^3^)† | Low | 582 | 98.072 | .15 | -33.787 | 229.932 | <.001 |
|  | High | 559 | -96.087 | .236 | -255.052 | 62.878 | .002 |
| *Note: ^#^ A median split in BMI-SDS is performed. Participants with a BMI-SDS ≤ 0.1164 are assigned to the low BMI-SDS subgroup and the others to the high BMI-SDS subgroup. Analyses are adjusted for age and ethnicity of the child and age, educational level and alcohol use of the mother during pregnancy. Unstandardized Beta’s are presented. †Analyses are also adjusted for total intracranial volume.* | | | | | | | |

| Supplementary table 5. The association between restrained eating subgroups and brain volumes in girls. | | | | | | | | | |
| --- | --- | --- | --- | --- | --- | --- | --- | --- | --- |
|  | **Split** | **Restrained eating subgroups** | | | | **df** | **F** | **η_p_^2^** | **P-value** |
|  |  | **Lower group** | | **Upper group** | |  |  |  |  |
| Outcome |  | **N** | **M(SD)** | **N** | **M(SD)** |  |  |  |  |
| Total cerebral white matter volume (mm^3^) | 20/80 | 910 | 403682.55 (3986  .49) | 232 | 406196.60 (38738.18) | 1 | 2.72 | .002 | .099 |
| Amygdala (mm^3^)† | Median | 654 | 1698.74 (156.97) | 488 | 1715.00 (171.98) | 1 | 5.42 | .005 | .020 |
| Hippocampus (mm^3^)† | 20/80 | 910 | 3912.21 (312.28) | 232 | 3943.96 (344.63) | 1 | 3.61 | .003 | .058 |
| Medial orbitofrontal cortex (mm^3^)† | 20/80 | 910 | 6212.42 (683.35) | 232 | 6267.91 (658.13) | 1 | 1.60 | .001 | .207 |
| Insula (mm3) † | Median | 654 | 7809.24 (788.48) | 488 | 7823.05 (768.49) | 1 | 1.37 | .001 | .242 |
| *Note: Restrained eating is split by the highest 20% versus the lowest 80% for linear models and by a median split for quadratic associations. Analyses are adjusted for age and ethnicity of the child and age, educational level and alcohol use of the mother during pregnancy. †Analyses are also adjusted for total intracranial volume. Effect estimates are based on ANCOVA’s with F=F-test of significance, df=degrees of freedom and η_p_^2^=partial eta-squared.* | | | | | | | | | |

| Supplementary table 6. The association between BMI-SDS and brain volumes in boys | | | | | | |
| --- | --- | --- | --- | --- | --- | --- |
| Outcome | **Model** | **B** | **P-value*** | **95% CI for B** | | **R^2^ change for BMI** |
|  |  |  |  | **Lower** | **Upper** |  |
| Total intracranial volume (mm^3^) | Quadratic | --6997.72 | .004 | -11723.26 | -2271.17 | .003 |
| Total gray matter volume (mm^3^) | Linear | 4091.59 | .008 | 1061.84 | 7121.33 | .007 |
| Total cerebral white matter volume (mm^3^) | Linear | 4189.98 | <.001* | 1750.54 | 6629.42 | .013 |
| Amygdala (mm^3^)† | Linear | 18.76 | <.001* | 10.09 | 27.43 | .009 |
| Cingulate (mm^3^)† | Linear | 104.01 | <.001* | 45.79 | 162.22 | .006 |
| Hippocampus (mm^3^)† | Linear | 25.22 | .003 | 8.52 | 41.91 | .001 |
| Lateral orbitofrontal cortex (mm^3^)† | Linear | 32.87 | .158 | -12.80 | 78.54 | .001 |
| Medial orbitofrontal cortex (mm^3^)† | Linear | 39.94 | .017 | 7.22 | 72.66 | .002 |
| Insula (mm^3^)† | Linear | 50.66 | .013 | 10.80 | 90.51 | .003 |
| *Analyses are adjusted for age and ethnicity of the child and age, educational level and alcohol use of the mother during pregnancy. Curve fitting results, in which p-values of all models per analysis are provided, are presented in supplementary table 3. †Analyses are additionally adjusted for total intracranial volume. *Analyses remain significant after Benjamini-Hochberg correction. B=Unstandardized Beta.* | | | | | | |

| Supplementary table 7. The significant associations between BMI-SDS subgroups and brain volumes in boys. | | | | | | | | | |
| --- | --- | --- | --- | --- | --- | --- | --- | --- | --- |
| Outcome | **Split** | **BMI-SDS subgroups** | | | | **df** | **F** | **η_p_^2^** | **P-value** |
|  |  | **Lower group** | | **Upper group** | |  |  |  |  |
|  |  | **N** | **M(SD)** | **N** | **M(SD)** |  |  |  |  |
| Total cerebral white matter volume (mm^3^) | 20/80 | 218 | 446155.98 (43103.33) | 888 | 451125.50 (45111.31) | 1 | 3.47 | .003 | .063 |
| Amygdala (mm^3^)† | 20/80 | 218 | 1819.7092 (169.63) | 888 | 1865.42 (187.99) | 1 | 10.21 | .009 | .001 |
| Cingulate (mm^3^)† | 20/80 | 218 | 12935.0367 (1403.63) | 888 | 13262.94 (1475.52) | 1 | 8.415 | .008 | .004 |
| Insula (mm3) | 20/80 | 218 | 8386.69 (904.62) | 888 | 8567.33 (857.76) | 1 | 7.28 | .007 | .007 |
| *Note: ^#^BMI-SDS is split by the lowest 20% versus the highest 80% for linear models and for quadratic associations a median split is performed. Analyses are adjusted for age and ethnicity of the child and age, educational level and alcohol use of the mother during pregnancy. †Analyses are also adjusted for total intracranial volume. Effect estimates are based on ANCOVA’s with F=F-test of significance, df=degrees of freedom and η_p_^2^=partial eta-squared .* | | | | | | | | | |

| Supplementary table 8. The association between restrained eating and brain volumes in boys | | | | | | |
| --- | --- | --- | --- | --- | --- | --- |
| Outcome | **Model** | **B** | **P-value** | **95% CI for B** | | **R^2^ change REB** |
|  |  |  |  | **Lower** | **Upper** |  |
| Total intracranial volume (mm^3^) | Linear | -512.20 | .454 | -1853.69 | 829.28 | .000 |
| Total gray matter volume (mm^3^) | Linear | -15.76 | .959 | -619.28 | 587.77 | .000 |
| Total cerebral white matter volume (mm^3^) | Linear | 234.46 | .344 | -251.30 | 720.21 | .001 |
| Amygdala (mm^3^)† | Linear | 1.17 | .187 | -0.57 | 2.90 | .002 |
| Cingulate (mm^3^)† | Linear | 5.18 | .384 | -6.47 | 16.82 | .000 |
| Hippocampus (mm^3^)† | Linear | .00 | .999 | -3.34 | 3.34 | .000 |
| Lateral orbitofrontal cortex (mm^3^)† | Linear | 2.70 | .561 | -6.40 | 11.80 | .000 |
| Medial orbitofrontal cortex (mm^3^)† | Linear | 1.71 | .609 | -4.82 | 8.23 | .000 |
| Insula (mm^3^) † | Linear | 1.51 | .94 | -40.05 | 43.07 | .000 |
| *Analyses are adjusted for age and ethnicity of the child and age, educational level and alcohol use of the mother during pregnancy. Curve fitting results, in which p-values of all models per analyses are provided, are presented in supplementary table 3. †Analyses are also adjusted for total intracranial volume. RE= restrained eating; B=Unstandardized Beta.* | | | | | | |
